# Internet of Things and New Technologies for Tracking Perioperative Patients With an Innovative Model for Operating Room Scheduling: Protocol for a Development and Feasibility Study

**DOI:** 10.2196/45477

**Published:** 2023-07-05

**Authors:** Eleonora Bottani, Valentina Bellini, Monica Mordonini, Mattia Pellegrino, Gianfranco Lombardo, Beatrice Franchi, Michelangelo Craca, Elena Bignami

**Affiliations:** 1 Department of Engineering and Architecture University of Parma Parma Italy; 2 Anesthesiology, Critical Care and Pain Medicine Division Department of Medicine and Surgery University of Parma Parma Italy

**Keywords:** internet of things, artificial intelligence, machine learning, perioperative organization, operating rooms

## Abstract

**Background:**

Management of operating rooms is a critical point in health care organizations because surgical departments represent a significant cost in hospital budgets. Therefore, it is increasingly important that there is effective planning of elective, emergency, and day surgery and optimization of both the human and physical resources available, always maintaining a high level of care and health treatment. This would lead to a reduction in patient waiting lists and better performance not only of surgical departments but also of the entire hospital.

**Objective:**

This study aims to automatically collect data from a real surgical scenario to develop an integrated technological-organizational model that optimizes operating block resources.

**Methods:**

Each patient is tracked and located in real time by wearing a bracelet sensor with a unique identifier. Exploiting the indoor location, the software architecture is able to collect the time spent for every step inside the surgical block. This method does not in any way affect the level of assistance that the patient receives and always protects their privacy; in fact, after expressing informed consent, each patient will be associated with an anonymous identification number.

**Results:**

The preliminary results are promising, making the study feasible and functional. Times automatically recorded are much more precise than those collected by humans and reported in the organization’s information system. In addition, machine learning can exploit the historical data collection to predict the surgery time required for each patient according to the patient’s specific profile. Simulation can also be applied to reproduce the system’s functioning, evaluate current performance, and identify strategies to improve the efficiency of the operating block.

**Conclusions:**

This functional approach improves short- and long-term surgical planning, facilitating interaction between the various professionals involved in the operating block, optimizing the management of available resources, and guaranteeing a high level of patient care in an increasingly efficient health care system.

**Trial Registration:**

ClinicalTrials.gov NCT05106621; https://clinicaltrials.gov/ct2/show/NCT05106621

**International Registered Report Identifier (IRRID):**

DERR1-10.2196/45477

## Introduction

Operating rooms (ORs) are responsible for a large amount of profit and cost [1]. About 60% of all hospitalized patients are treated in the OR [2]. Surgical scheduling is a key process in perioperative organization; it begins by analyzing a list of daily cases and their expected duration. If cases consistently run longer, OR overuse will result in costly overtime pay and staff dissatisfaction. On the other hand, if actual case times are shorter than expected, OR underuse will lead to staff idle time, which is associated with up to 60% higher costs [3-5]. Also, decisions about the number of surgeries to perform during a typical day or week of activity in the OR have a considerable impact on downstream resources and on the efficient flow of patients in the system. Combining higher-level decisions, optimization logic, and management policies with an analysis of the consequences of these decisions that is enabled by simulation tools is an effective approach in health care [6-8]. Also, simulation is useful for testing the expected performance of a process, as well as its performance when it has been revised, modified, or optimized, before implementing it in practice and can also evaluate unexpected scenarios. Case duration prediction is usually demanded by the surgeon who reserves the time slot. With this method, it has been proven that surgeons underestimate case duration up to 42% of the time and overestimate it 32% of the time [9]. Another common approach is to use the electronic health record (EHR) to calculate case duration based on historical data for a given procedure. Decisions based on EHRs have a higher accuracy [10], but available EHRs only generate case durations for the average patient and do not take into account the patient’s specific profile (eg, their age, sex, BMI, allergies, or comorbidities). The rise of big data offers novel possibilities for patient-targeted predictions by exploiting machine learning (ML) and deep learning (DL) techniques. In particular, supervised learning techniques are able to identify latent patterns among large quantities of data by training the model to minimize a loss function between the actual output and the desired (ie, supervised) one. The model is afterward able to generalize on unseen cases that belong to the same data distribution. For this reason, ML is also gathering attention in the medical field for both clinical and organizational purposes. Notwithstanding this, we are still in the early phase, and there are several challenges to deal with [11-14] .The main issue to exploit ML is providing accurate and noise-absent samples to the model, which is critical when historical data are collected by human processes. In light of this, the aim of our research project is to develop an integrated technological-organizational model capable of exploiting data derived from ORs to optimize the management and organization of the whole operating block. To achieve such a result, we have first developed an internet of things (IoT) architecture that is able to collect real data to minimize the presence of errors or noise in data and maximize ML performance. Next, we will proceed to develop an optimal scheduler for surgical procedures supported by simulation and able to integrate clinical and anamnestic information with data derived from an analysis of surgical timing and time spent in the recovery room (RR) so as to optimize the system’s organization [15].

## Methods

### Overview

Our research work is a prospective, single-center, interventional study. Patients are included if they are older that the age of majority (18 years in the country where the protocol was drafted); provide signed informed consent; and are undergoing elective abdominal, thoracic, vascular, gynecological, or plastic surgery. If the patient agrees to participate in the study, data will be collected on the expected and actual time from the patient’s entry to the operating compartment to their exit. In particular, time spent in the operating room, surgical time, and length of stay in the operating room will be recorded. This information will be integrated with anamnestic data and data relating to hospital stay [16-18]. The goal is to create a technological-organizational programming model able to optimize operating block management. In this work, we identified 50 simple variables for each patient. These features will form a single data set instance. We decided to focus our attention on ML methods instead of traditional methods, as the latter may not be sufficient for this task, often requiring assumptions about data distribution, and may not be able to handle the complexity and volume of the data that we want to use to make predictions. On the other hand, ML methodologies can capture nonlinear relationships between variables, which can be important for predicting times accurately. Moreover, ML algorithms can benefit from large amounts of data to identify patterns, and EHR data contains a large number of variables, including patient anamnestic data, medical records, and surgical details. ML algorithms can extract knowledge from this data and use it for making a much better and more accurate prediction. Finally, ML methods can handle outliers, which can be common in EHR data, and ML methods can generalize well to new data. This is important because we can make predictions for surgery time for patients who are not in the original data set. In this study, we will collect data from 1200 patients. Bearing in mind that this data set will have to provide data to both create the model and validate it, the data set will be divided into 3 parts: a training set (60%), a validation set (20%), and a test set (20%). The project is divided into three phases: (1) using the IoT for data collection and patient enrollment, (2) machine learning for time prediction, and (3) optimization with discrete-events simulation.

In the first phase, which forms the focus of this paper, we developed an IoT architecture to perform indoor localization of patients in ORs and in RRs. Patients who decide to participate in the study are asked to give informed consent. If they agree, they are provided with a personal Bluetooth Low Energy (BLE) beacon tag when entering the operating block or in the ward. The tags are detected by detectors (Raspberry Pi devices; Raspberry Pi Ltd) that are located in the environment of interest. The detectors communicate with a private local area network (LAN) to manage data flow and to provide additional security levels. Moreover, the LAN does not have any internet access, so the network is not vulnerable to any external attacks. The access to the server, to the sensor configuration module, and to the association interface is password protected and all communication to the server is encrypted. In this section, we describe the components and our solution for monitoring patients’ movements inside the surgical block. We realized a client-server architecture that provides communication between the sensor modules and the server in our system. The whole IoT system has several parts, which are listed below.

### BLE Beacons

BLE is a wireless communication technology designed for low-power devices that require short-range connectivity and is widely used in IoT environments [19]. BLE technology operates on 2 main channels: advertising and data. For our purposes, we decided to use only the advertising channel in order to detect beacons when they are near to our sensors. For this reason, we adopted the iBeacon protocol (Apple Inc). The iBeacon protocol identifies beacons with a triplet of universally unique identifiers (UUIDs). For this prototype we used the BlueUp (BlueUp Srl) beacon, which has 800 milliseconds of advertisement time. Each patient will be associated with a beacon connected to a numerical, progressive, and anonymous ID to respect patient privacy. The beacon is assigned to each patient and is worn as a bracelet with a wrist band. Being affixed to the patient’s wrist is the best compromise between comfort and signal efficiency.

### Sensor Modules

As a sensor module, we used a Raspberry Pi 4 device running the 64-bit Raspian OS. The sensor module continually scans for BLE advertisements, checking them against a list of preregistered beacons provided by the central server. Using a list allows us to ignore nonregistered beacons, saving bandwidth and processing resources. The sensors autoconfigure themselves. They can be rebooted or shut down at any time. When the sensors receive a beacon advertisement, they append the package received and its timestamp into a JSON file and forward the information to the central server over a transmission control protocol.

We estimate the patient’s presence inside a specific room in the operating block using the residence time within the range of action of the sensor. Every sensor has its own residence time and a tunable threshold of signal sensitivity. This last parameter can increase or decrease the sensor’s range of action. If the beacon remains for a certain amount of time in the action range of a sensor, the beacon is marked as entered into the room.

### Central Server

The central server indexes and collects the data coming from the sensor modules. It has the following duties: (1) storing records coming from each detector in a MongoDB database (MongoDB Inc); (2) coordinating distributed solution and message exchange using a publish-subscribe mechanism based on message queue telemetry transport (MQTT), and finally, (3) exposing a web server that is used as a unique interface between the software architecture and the hospital medical staff. The central server hosts the eclipse Mosquitto-based MQTT broker (Eclipse Foundation Inc). When it receives the packets from the sensors, it determines whether the beacon is in the room where the sensor is located. Our implementation of the beacon server used a Python-based service and exploited the MongoDB database to store the various events. The data format is shown in Table 1. The central server also has the duty to send information to the edge modules regarding the list of preregistered beacons, the identity of the sensor itself, and a time-synchronization message.

**Table 1:** Data stored by the server for each sensor when an entry is recorded in the database.

Fields	Description
uuid	Universally unique identifier for the beacon
minor	Contains the unique beacon ID
macAddress	Beacon MAC address
timestamp	First detection timestamp
last_detection	Last detection timestamp (continuously updated)
flag	Boolean value that if true specifies that the patient is currently in that specific room
Patient ID	Unique number assigned to a specific patient

### Client Interface

**Figure 1:**
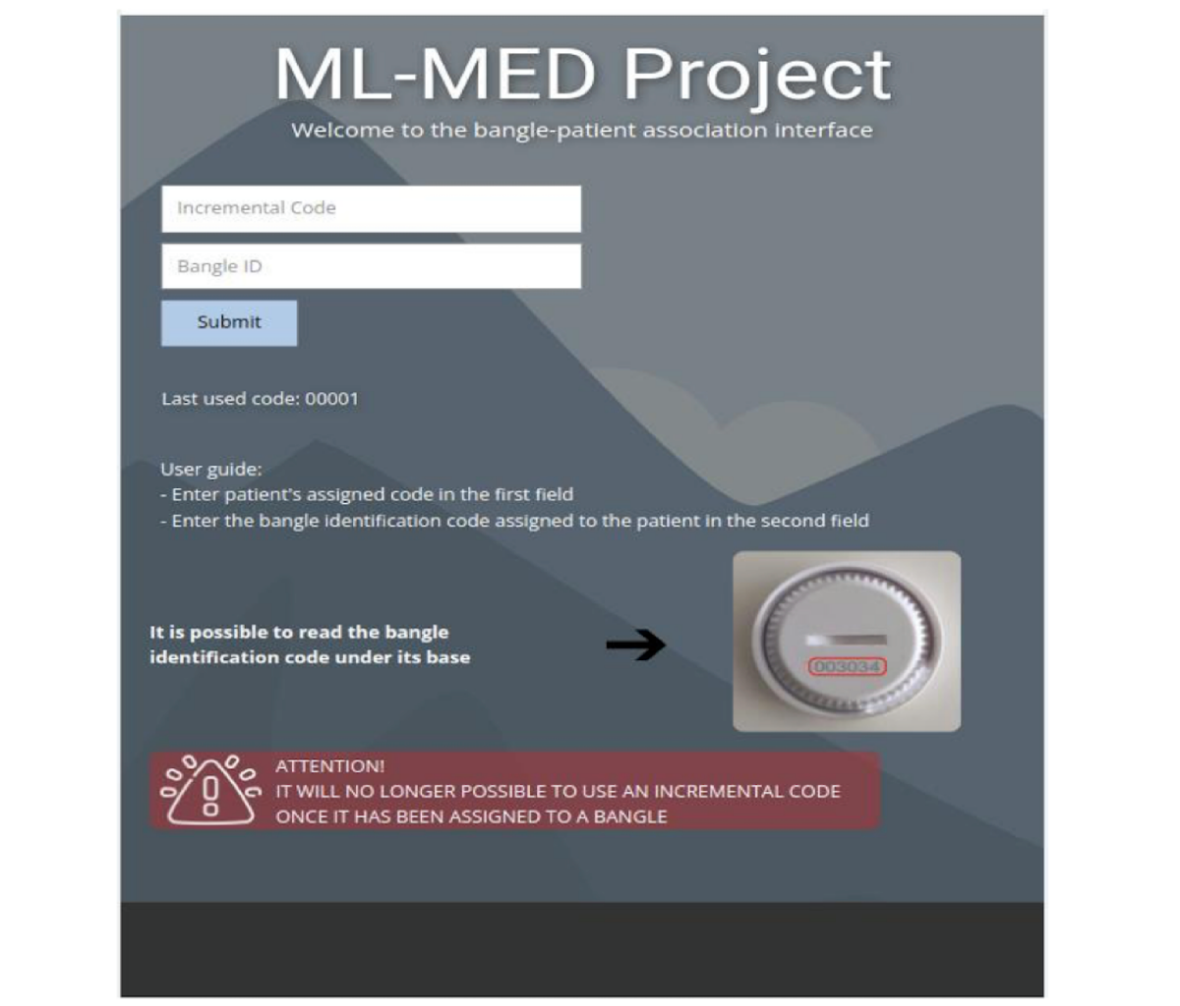
Screenshot of the home page of the web application responsible for the beacon-patient association. (In the web application, the term “bangle” is used to describe the beacon.).

**Figure 2:**
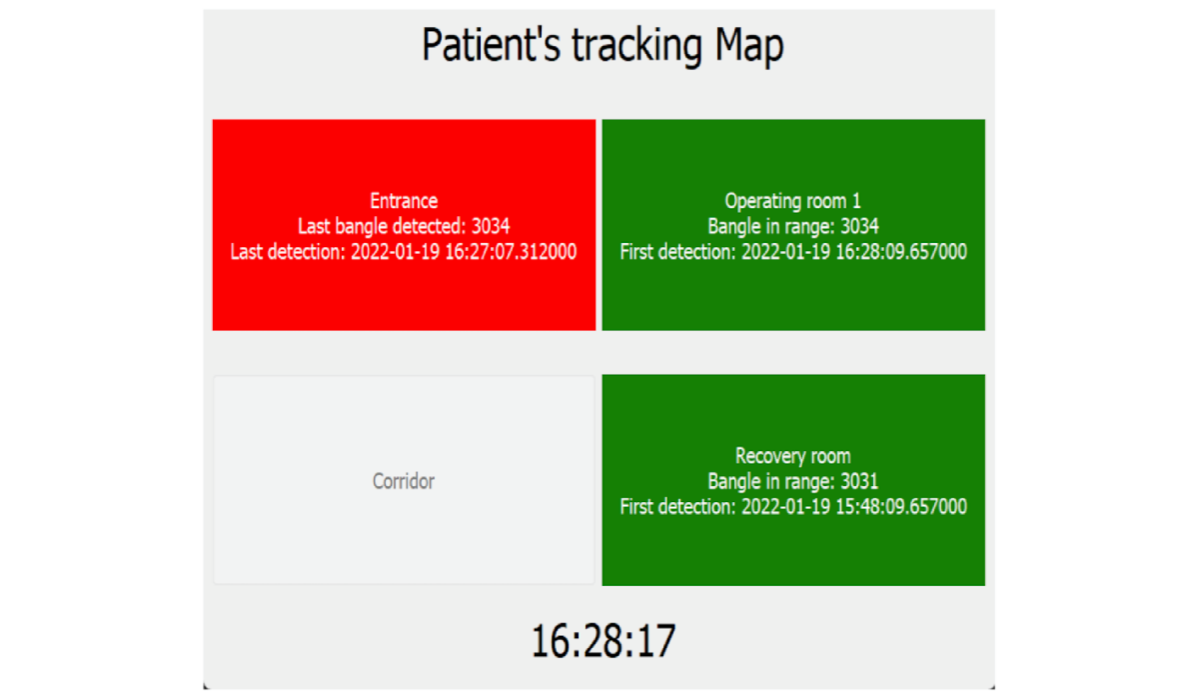
Screenshot of the graphical interface for monitoring patient movements inside the operating block. Green cells indicate that the patient is near the sensor that is associated with that specific room or place. Red cells indicate that no tracking device has been detected for some time. Finally, gray cells indicate that there is no tracking data for that place.

The client interface, hosted by the central server, offers several functionalities. The first is enrollment. Patient enrollment is done by a member of the medical staff when the patient enters the operating block to undergo surgery. The enrollment is performed using a tablet device that communicates with the web application and by providing the necessary information. The system also provides a graphical interface able to monitor in real time the various movements of patients (Figures 1 and 2).

### Ethics Approval

The study was approved by the Comitato Etico Unico per l’Area Vasta Emilia Nord, a local ethics committee (1284/2020/OSS/AOUPR), and was registered on ClinicalTrials.gov on January 11, 2021 (NCT05106621). Written informed consent was obtained for all study participants. The participation of individuals of legal age capable of giving informed consent as subjects in medical research must be voluntary (World Medical Association Helsinki Declaration). The data are collected anonymously and with respect for the privacy of patients.

## Results

This research was financially supported by the FIL-Quota Incentivante program of the University of Parma and cosponsored by Fondazione Cariparma with approval in the second half of 2019. As of February 2023, approximately 1000 patients have been enrolled, with an enrollment target of not less than 1200 to 1300.

**Figure 3:**
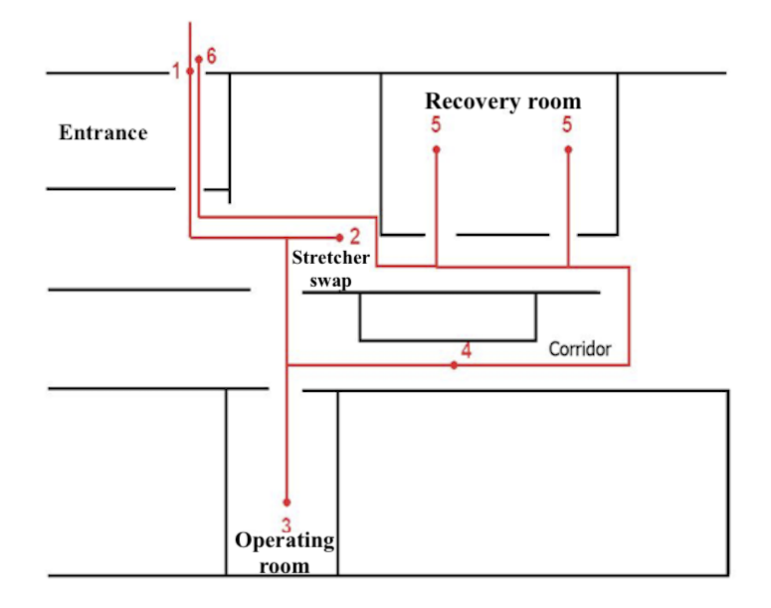
Generic patient path illustration within the operating block. The patient accesses the operating block at tracking point 1. Then, the patient’s stretcher is swapped at point 2, and a member of medical staff assigns them a beacon and sets up the associated ID. Next, the patient goes to the assigned operating room (point 3). Once the surgical procedure ends, the patient is transferred to the recovery room (point 5), where they are monitored after the procedure. The patient can then either exit the operating block from point 6 or can return to the operating room if there are acute surgical complications. Moreover, during these activities, the patient can remain idle at an unspecified point in the corridor, represented by point 4. Personal data are extracted from the information systems of the Ospedale Maggiore of Parma using pseudoanonymization techniques.

**Figure 4:**
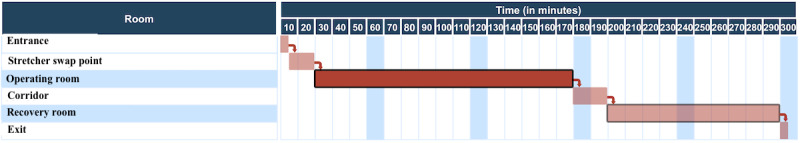
Visualizing patient flow with a Gantt chart showing average time spent by patients in each room over time.

**Figure 5:**
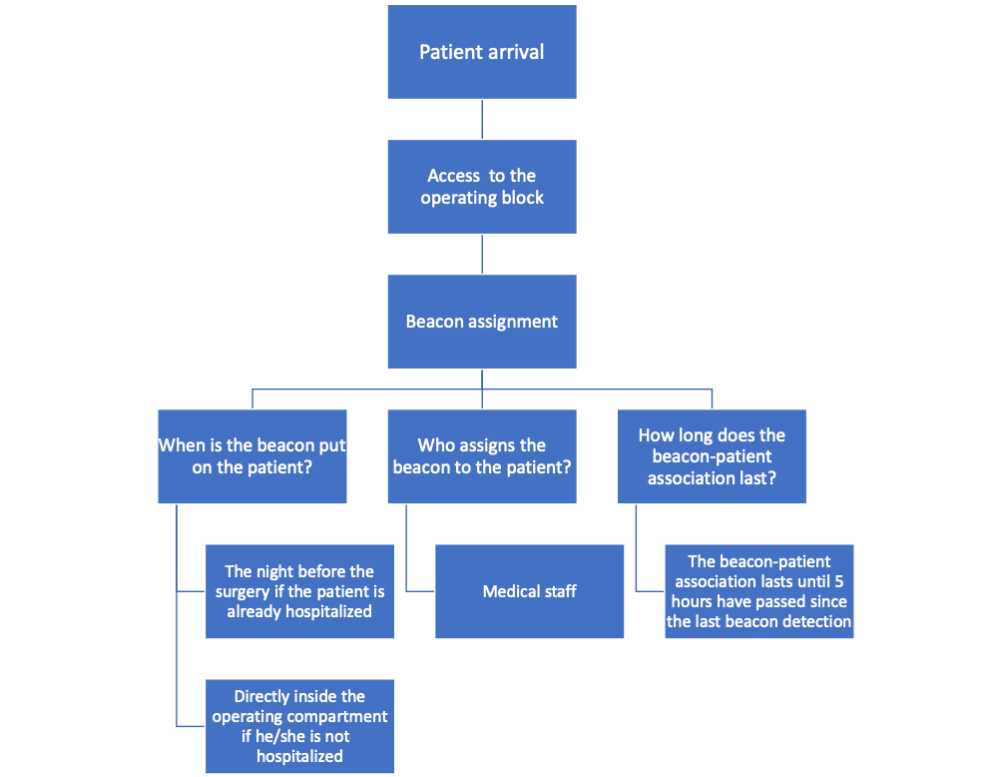
Easy identification of the patient in the operating block through the association with a tag and a code.

**Figure 6:**
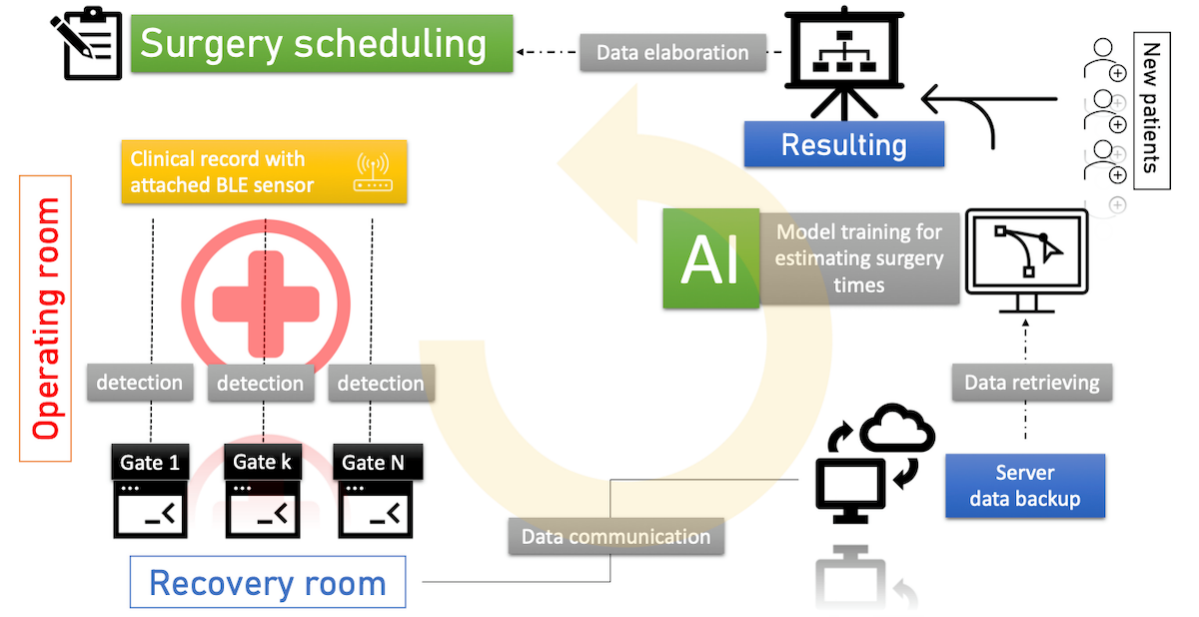
Logical schema of the indoor patient tracking system, data storage, and data set analysis using machine learning models. Data security is ensured by different levels of protection at different steps. AI: artificial intelligence; BLE: Bluetooth Low Energy.

We schematized a generic path a patient could follow inside the operating block (Figures 3-6).

In addition, if a patient agrees to participate in the study, we collect the following: data about the surgical procedure, personal and anamnestic data, data from the RR, and data on the hospital stay, such as whether the patient was admitted to the intensive care unit.

The prototype of the IoT architecture described and illustrated above was installed successfully in the surgical block and is still functional. We collected data from more than 100 patients. It is possible to see in the final output illustration (Table 2) that combining the data from various sensors allowed us to reconstruct the patient’s path.

The second step will involve the application of ML and DL algorithms to study the feasibility of predicting surgical procedure time by providing features of the patient and of the surgery as input. Because of the lack of public data sets or similar approaches in the scientific literature, we are currently performing a feature selection task among more than 50 variables to identify which features can maximize prediction accuracy in terms of the root mean squared error of surgery time. Several ML approaches are possible since the task of intraoperative time prediction can be modeled in different ways. Since the total time in the operating block is the sum of the time spent in the OR and in the RR, this prediction task can be modeled using 2 different ML models or a unique one that tries to directly estimate the total time. Moreover, depending on the data set size, tree-based ensemble models (eg, gradient boosting or extreme gradient boosting; XGBoost) could better suit the task. On the other hand, the complexity of the task and the nonlinear dependency of several features within the target variable of “time required” may require the use of deep neural networks. DL requires larger data collection, but at the same time, it would be possible to exploit its ability to perform representation learning of the input, requiring less feature engineering.

**Table 2:** The patient’s path within the operating block with the relative timestamps and time spent in every room.

Beacon ID	Patient ID	Room sensor	First timestamp	Last timestamp	Total time
3287	PR-0170	Entrance	2022-05-24 07:47:31.988000	2022-05-24 07:51:18.086000	00:03:46.098000
3287	PR-0170	Corridor	2022-05-24 07:51:50.639000	2022-05-24 07:53:03.557329	00:01:12.918329
3287	PR-0170	Operating room	2022-05-24 07:54:52.586975	2022-05-24 10:59:05.650000	03:04:13.063025
3287	PR-0170	Recovery room	2022-05-24 11:00:08.795296	2022-05-24 12:54:34.885000	01:54:26.089704
3287	PR-0170	Entrance	2022-05-24 12:54:44.533703	2022-05-24 12:57:04.107000	00:02:19.573297

Once ML is trained to predict the surgery time, we will proceed to step 3, which is the use of simulation models of discrete events. In these models, an “event” is an instantaneous occurrence that involves a change in the value of at least one of the system variables; for instance, it could be the entrance of a new patient, the exit of a patient from the system, or the transition of the patient from one room to another. The goal of the simulation model is to reproduce the patients’ path within the operating block (including the preoperative, operative, and postoperative phases) to optimize their flow in the system. To this end, some selected key performance indexes will be embodied in the simulation model; examples of these indexes are the number of patients treated daily, use of the OR, and the need for extra time working in the OR. Simulation will be first used to evaluate these indexes in the current scenario (“as is” analysis) then to reproduce new logic for scheduling surgical interventions (“to be” re-engineering) based on multiple prioritization logics or multi-objective optimization. The improvement achieved in the performance indexes will also be evaluated [20].

## Discussion

### Principal Findings

The use of artificial intelligence (AI), and in particular ML, is expanding in every area, including health care. Many results are now available on the excellent predictive capabilities of these new tools in medicine. Thanks to their use, intelligent tools useful to support health care professionals in daily practice will be increasingly available. Their possible exploitation in the health organization is no exception. Liu et al [21] showed that ML is superior to logistic regression for risk estimation in the context of hospital performance assessment. Furthermore, similar applications can be found in the context of forecasting health care costs, risk of readmission, and hospitalization [21-24].

The study by Luo et al [24] is also very interesting; ML models were applied with the intent of estimating the risk of cancellation of an operating session with the negative impact that this entails both in terms of costs and on waiting lists; therefore, cancellation also translates into delayed access of the patient to surgery. Moreover, a novel approach has been presented by Abbou et al [25]. The authors gathered EHR data from December 2009 to May 2020 for a total of 297,480 interventions at 2 public hospitals in Israel. They predicted the duration of the surgery based on preoperative data, including patient clinical data, the experience of the surgeons, patient nationality, and results of analyses carried out before the operation. They compared the predictions between a naive model and an ML model (XGBoost) with various metrics and found that the use of big data was useful for predicting the duration of interventions in the operating room and that the ML model performed better than their naive model.

However, to apply these models, it is essential to obtain data with quality and precision. Currently, recording times and patient movements within the surgical block is often done manually by the various medical staff involved and subsequently reported in computer systems. However, this detection method is often partial and usually not done in real time. The possibility of enabling direct recording with minimum human interference could increase the quality of the data set and let us obtain more precise results [26-28]. Furthermore, having a system equipped with the ability to independently record the patient’s movements, besides reducing the error rate, could allow for lightening the workload of the medical staff themselves and indirectly reducing the changeover time between different patients [24,28-32]. Nevertheless, building a tracking system inside a hospital is not a simple task. Not all hospitals are equipped with the highest technology available. Some hospitals are small and do not have sufficient resources available to install high-technology organizational systems. Others, despite being larger in size, have old structures in which it is not always easy to implement new technologies. Therefore, we decided to look for a solution that suits our needs and is economical, does not require an expensive infrastructure, and is achievable in most situations.

To understand which technologies were the best for our use case, we decided to analyze several options and constraints. The main constraints encountered were ease of installation, the tracking device’s battery life, and the tracking device’s reuse and cleaning.

Considering these major constraints, we analyzed and considered several technologies. The first was radio frequency identification (RFID); unfortunately, this technology had a cumbersome structure for tracking and the range of action was limited, which could have brought discomfort to the members of the medical staff. Moreover, the RFID device’s battery life was not sufficient for our case study. Next, we analyzed ultrawideband (UWB), which was discarded as well due to the low autonomy of the devices. Finally, we decided to use a BLE tracking device.

BLE fitted our use case because the detectors are small and easy to install, the device’s range of action is very wide, and the tracking device’s battery life can last several months. Furthermore, our use case does not involve tracking health care personnel, only patients.

Building a tracking system using BLE also has economic advantages; it does not burden the hospital budget and is cost-effective. The system presented above can trace the movements of patients within the OR. The data obtained will form a data set that can be used to perform ML tasks. By combining the tracking data with clinical assessment data, it will be possible to create an algorithm capable of predicting the duration of a specific surgical intervention.

The IoT architecture developed in this study will be useful to collect real data about patient flow in the surgical department. Nonetheless, having solid data and predictions about OR occupancy is still not enough. It is also necessary to create organizational systems capable of using that information. In fact, not being able to transform these results into intelligent tools that can be used in daily practice has been recognized as one of the major limitations that currently hinder the use of these technologies in medicine [33]. To overcome these limits, a simulation model will be developed for reproducing patient flow in the operating block. This model, fed with high quality data, will be used to determine the best scheduling logic in order to optimize the use of resources in the OR. Preliminary testing of the model was performed using randomly generated patient data in an attempt to check its effectiveness in reproducing the system, as well as to embody alternative scheduling logics and return the performance indexes as outcomes. Results, both in terms of the model capability to reproduce the current system and in evaluating its performance, are promising and at the same time highlight the potential for improvement in the efficiency of the surgery department, which is the ultimate aim of the project. In a more advanced phase of the research, the simulation and scheduling logics will be enhanced and further improved by the availability of accurate predictions enabled by an ML model. In general terms, it is expected that an accurate evaluation of patient flow (based on real data) and a reliable prediction of surgery cases (based on the ML model) will make it possible to optimize planning of surgical procedures, consequently decreasing unused OR time and hopefully increasing the number of surgical interventions in a day. This is an innovative aspect of the whole project; indeed, although ML, simulation, and scheduling have been applied in health care environments, their combined use opens new ways for process improvement. This approach, thanks to its consistent predictive performance over various forecast intervals, can positively influence the choices of health care personnel for short-term and long-term strategic planning [34-36].

### Conclusions

**Figure 7:**
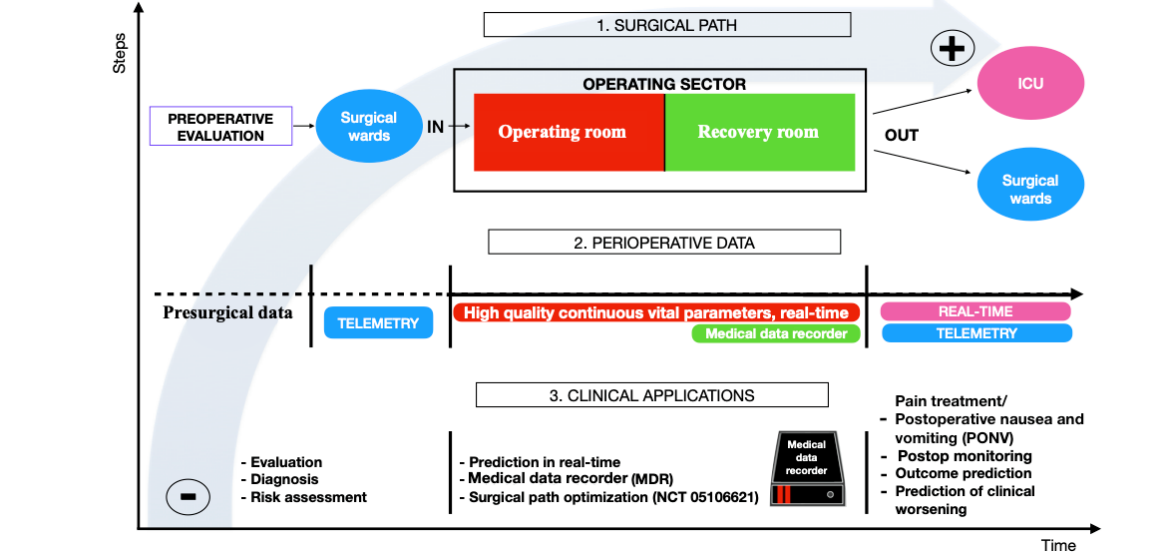
Use of artificial intelligence in the operating sector for a more targeted organization of resources. ICU: intensive care unit.

Surgery has a great impact on the health economy; thus, optimal management of resources destined for the OR becomes crucial. Considering the literature and our preliminary results, it therefore seems possible to assume that the application of AI models in the context of OR organization, associated with an indoor patient traceability system, is not only feasible but could also lead to more targeted organization (Figure 7) [37,38].

## Abbreviations

AI: artificial intelligence
BLE: Bluetooth Low Energy
DL: deep learning
EHR: electronic health record
IoT: internet of things
LAN: local area network
ML: machine learning
MQTT: message queue telemetry transport
OR: operating room
RFID: radio frequency identification
RR: recovery room
UUID: universally unique identifier
UWB: ultrawideband
XGBoost: extreme gradient boosting

## Data Availability

The data sets used and/or analyzed during the current study are available from the corresponding author on reasonable request.
